# They Will Never Forget How You Made Them Feel: Implementing Harm Reduction in the Perinatal Setting

**DOI:** 10.1007/s10995-023-03795-1

**Published:** 2023-11-02

**Authors:** Joelle Puccio

**Affiliations:** Academy of Perinatal Harm Reduction, 2850 SW Cedar Hills BLVD #2061, Beaverton, OR 97005 USA

**Keywords:** Pregnancy, Substance Use, Perinatal Harm Reduction, Harm Reduction

## Abstract

Harm reduction is a framework built upon respect for people who use drugs. Pregnancy is a priceless window of opportunity for positive change, when parents are driven to improve their health and well-being for their future child, and harm reduction can provide direction for this motivation. Perinatal harm reduction can include goals of abstinence, decreased use, safer use, or a goal unrelated to substance use, such as obtaining housing or employment. We engage in harm reduction, not only by promoting beneficial practices, but by eliminating harmful ones. Despite the science, effective program models, and overwhelming agreement that substance use disorder is a health condition and not deviant behavior, harm reduction programs for pregnant and parenting people are rare, and punitive treatment is the norm. To achieve equitable treatment for people with perinatal substance use disorder and protect the parent-infant dyad, we must stop harmful practices, respect the autonomy of people who use drugs, and address substance use disorders while increasing access to the social determinants of health.

## Introduction

**Figure:**
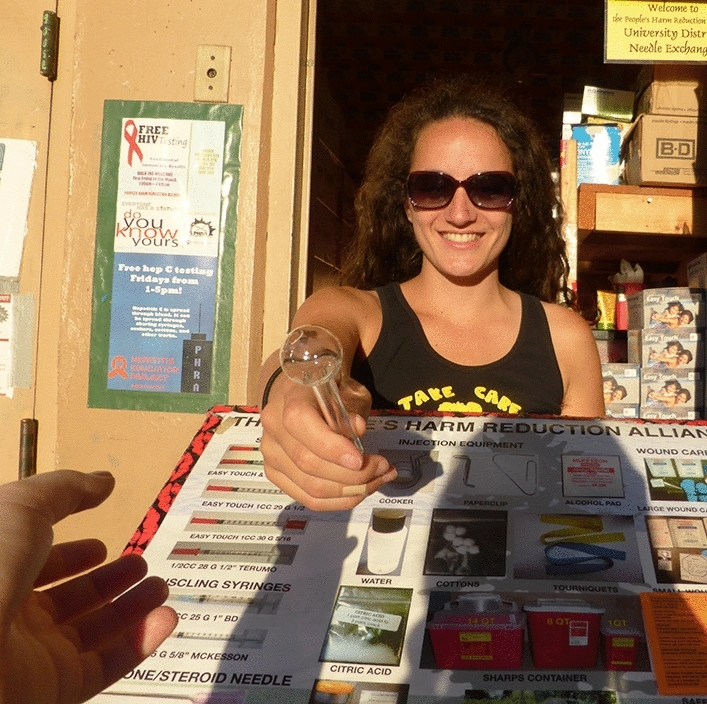
Outdoor photo in an alley on a sunny day. Joelle Puccio (they/them) is a white nonbinary person with long curly brown hair and sunglasses. They are wearing a black tank top with the words “take care” in yellow letters. Joelle is working at the People’s Harm Reduction Alliance (PHRA) in Seattle, WA. PHRA is a drug-user run, low-barrier syringe service program that offers injection, smoking, and snorting equipment. They are standing behind the syringe services menu, which shows pictures of various needles and other equipment. Joelle is smiling and offering a new meth pipe to someone whose hand is visible in the bottom corner of the photo. Photo courtesy of Joelle Puccio, taken by J. K. in 2017

I come to this topic both as a nurse and as someone who uses drugs. Both identities contribute to my role as a subject matter expert. Most often, I use legal substances such as alcohol because they are convenient to access. I see all substances as morally inert, without values attached. Caffeine, cocaine, and methylphenidate (Ritalin) are all stimulants used in numerous settings for various purposes. My perspective on substances allows me to avoid blaming people who use them and identify the legitimate root causes of health and social challenges associated with substance use.

Although my status as a person who was assigned female at birth and who uses drugs has made me vulnerable to incarceration and other barriers to employment, education, and housing, the story of my substance use is one of overwhelming privilege. As a white, able-bodied, neurotypical, middle-class, educated, childless professional, I can manage my substance use in ways most people cannot. However, my work must continue to be informed not only by my own experience, but by actively and humbly listening to the experiences of people different from myself. To paraphrase Audre Lord, “I am not free while any person is unfree, even when their shackles are very different from my own.” Any effort to eradicate racism, misogyny, or ableism must consider how these and other oppressions intersect for pregnant patients who use substances.

## Perinatal Substance Use

Overdose has been identified as a leading cause of death in the perinatal period (Bruzelius & Martins, 2022). Overdose death risk is amplified by stigma, which causes people to hide their substance use, preventing loved ones from responding to overdose (Thompson et al., 2023). Stigma and well-founded fear of punitive interventions have been identified as barriers to prenatal care and substance use disorder treatment (American College of Obstetrics and Gynecologists, 2011; Volkow, 2023).

It is critical that healthcare workers understand the effects of substance use on the pregnant person, fetus, and newborn in order to provide respectful and effective harm reduction (Goyer et al., 2020). Many of the risks associated with stigmatized or criminalized substance use are confounded by lack of access to the social determinants of health, such as access to prenatal care (El-Mohandes, 2003). Despite this, many healthcare workers prioritize educating patients about the risks of substance use above all else, but this is usually not the client’s greatest need. Patients are already acutely aware of the risks of substance use, as well as the stigma associated with it in society. Nobody wants to be a pregnant person who uses drugs chaotically. However, this is a situation in which some of us find ourselves. While most people stop or reduce their use when they become pregnant (SAMHSA, 2020), a small percentage are unable to do so. These people may have a substance use disorder, and they should be celebrated for showing the strength and vulnerability required to ask for help.

Pregnancy is a priceless window of opportunity for positive change. During this time, parents are motivated to improve their health not only for their own well-being, but for their future child. Harnessing this motivation requires health care providers to engage patients with empathy, creativity, and evidence-based, family-centered care. Perinatal harm reduction can provide direction for health care providers to help parents channel their motivation.

## What is Perinatal Harm Reduction?

The most basic definition of harm reduction is anything that reduces harm or promotes joy. The National Harm Reduction Coalition defines harm reduction as a set of practical strategies and ideas aimed at reducing negative consequences associated with drug use and a movement for social justice built on a belief in, and respect for, the rights of people who use drugs (National Harm Reduction Coalition, 2020). Examples of harm reduction with which most people are familiar are seatbelts, condoms, designated drivers, and insulin. In addition to promoting beneficial practices, we engage in harm reduction by eliminating harmful practices such as separation of children from parents who use substances in the absence of other risk factors. Harm reduction is effective, cost-effective, preventative, and a framework built upon respect for people who use drugs.

People who use drugs conceived every harm reduction intervention we rely on today. These practices were adopted by mainstream health and social services only after our forebears risked social stigma, arrest, and other forms of systemic violence for taking care of themselves and their communities. With regards to substance use, harm reduction is commonly associated with the distribution of naloxone kits, syringe services programs, medications for opioid use disorder, medication lock boxes, and blood borne infection testing.

Harm reduction for substance use is a practice with which every perinatal health care worker is familiar. For example, when we help our patients manage smoking during pregnancy, we encourage them to quit or cut back, avoid smoking near children, and support human milk feeding. When we view tobacco and other substance use through a harm reduction lens, we can promote safety, autonomy, and nurture our therapeutic relationships with patients. Just as we would never refuse care to a patient who is unable to quit smoking, it is counterproductive to abandon patients who do not achieve sustained abstinence from other substances.

**Fig. 1:**
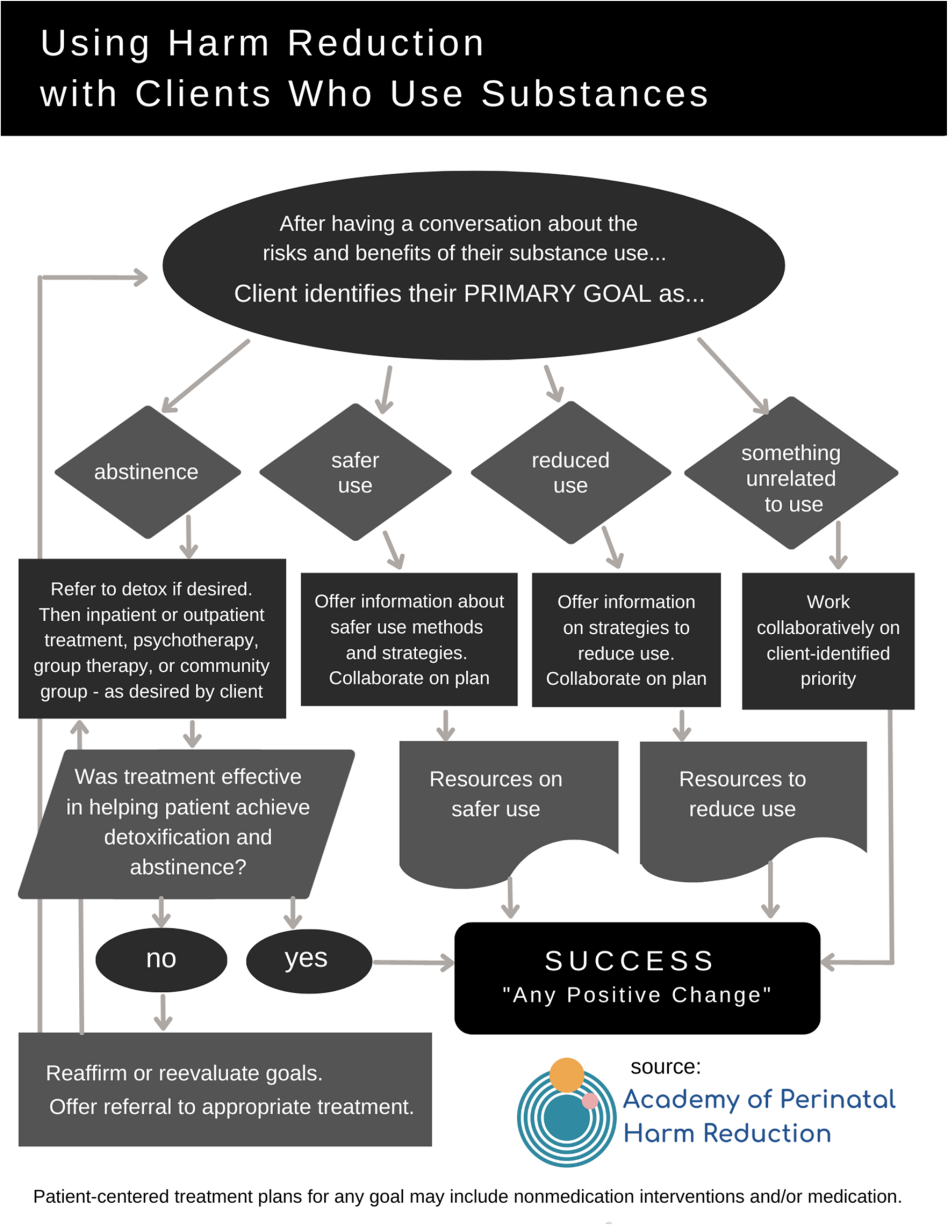
Navigating harm reduction service delivery

Perinatal harm reduction can include goals of abstinence, decreased use, safer use, or a goal unrelated to substance use, such as housing or employment. For example, a patient may plan to switch from injecting to the safer method of smoking, reduce their use to weekends only, set a goal to achieve abstinence by a certain date, and connect with a social worker to enroll in health insurance. They may work on these goals one at a time or simultaneously. Developed by the Academy of Perinatal Harm Reduction, Fig. 1 is a flowchart that shows how health care practitioners navigate harm reduction service delivery while promoting safety, autonomy, and therapeutic relationships with their patients.

## Perinatal Harm Reduction is a Critical Component of Reproductive Justice

Effective harm reduction in the perinatal period must anticipate the intersecting identities of parents, children, and families so that health care practitioners can protect against oppression in all its forms. Birthing people may simultaneously face misogyny, racism, and stigma related to having a substance use disorder. For example, beginning in the 1980s, the so-called “crack baby” scare coincided with the racist stereotype of the “welfare queen” as an unfit mother. Black families were disproportionately punished by the resulting laws and policies, despite similar rates of cocaine use to whites (Roane, 2015). Black, Indigenous, and women of color responded to these and other grave injustices by developing the Reproductive Justice framework, which supports the human rights of birthing people to own their bodies and control their futures, to have children, not to have children, and to parent the children they have in safe and sustainable communities (SisterSong, 2023).

## Addressing Harm Reduction Misconceptions

Harm reduction remains a controversial term. Misconceptions about harm reduction are rooted in stigma toward people who use substances.

### Harm Reduction Does Not Equate to Condoning Substance Use

Harm reduction is not focused on whether people should or should not use substances, but simply acknowledges that they do. Perinatal harm reduction is often met with skepticism by those who see it as allowing substance use in pregnancy. A more accurate conceptualization is that it allows pregnant people who use substances to seek help without judgement and punitive responses. Although many punitive interventions, such as mandated reporting laws, are well intentioned, they can result in lack of access to prenatal and other healthcare due to justified fear of criminal-legal system involvement or loss of child custody (American College of Obstetrics and Gynecologists, 2011; Volkow, 2023).

### Harm Reduction is Not a Uniform Approach for All Individuals and Communities

To create systemic change, we need to move away from the one-size-fits-all approach to harm reduction. Even for evidence-based approaches such as syringe services, every community requires a nuanced approach that addresses the unique needs of their populations who use substances. For example, although both require clean and sterile injection supplies, pregnant teens who use heroin require different harm reduction interventions than athletes who use steroids.

### There is No Need to Wait Until We Find Perfect Solutions to Abolish the War on People Who Use Drugs

A common barrier to implementing harm reduction is the notion that we must have proven replacement practices before we begin removing those that are harmful and ineffective. There is no reason to delay the elimination of harm while we seek a perfect response. For example, we can stop arresting people for drug possession immediately *and* invest resources in community programs aimed at treatment, employment, after-school programs, and overall neighborhood improvement. Furthermore, sometimes harmful practices affecting people who use drugs can be simply replaced with routine care. For instance, we can stop reporting parents to child services for taking their prescribed methadone and replace this policy with the routine care already provided to parents who take other prescription medications. As care plans, services, and programs are developed, evaluated and refined to meet a specific need, the affected and surrounding communities should be continuously involved.

## The State of Perinatal Care for People Who Use Substances

Despite the science, effective program models, and overwhelming agreement that a substance use disorder is a health condition and not deviant behavior, harm reduction programs for pregnant and parenting people are rare, and punitive treatment is the norm. As a travel nurse, I have worked in 14 hospitals in eight states within the last seven years. I have witnessed the following punitive practices in my professional career:

- Hospital units that impose limited or supervised visitation for parents and caregivers who currently use substances or have used them in the past.
- Exclusion of parents and caregivers who use substances from newborn care planning.
- Retaliatory child welfare reporting of parents and caregivers who attempt to contribute to newborn care planning.
- Exclusion of family members from local charitable accommodations if their newborns experience opioid withdrawal, even if the birthing parent was in treatment during pregnancy.

These actions trample on the basic human rights of people who use drugs. They are often contradictory to or omitted from official hospital policy. They are also discriminatory and possible violations of the Americans with Disabilities Act.

## Recommendations for Providers, Health Systems, and Policymakers

### First, Do No Harm

Access to quality perinatal care can be improved by simply treating patients who use substances in the same way we treat other patients. We must play a proactive role in eliminating harmful and ineffective provider and health system practices connected to the criminal-legal systems that negatively impact pregnant people and the parent-infant dyad. Policymakers at every level must call upon the expertise of healthcare workers and affected populations to ensure that parental substance use is not considered child abuse or neglect in all U.S. jurisdictions. Mandating healthcare workers to report substance use even when there are no concerns for child safety causes adversarial relationships between providers and patients, hindering care quality and the likelihood the patient will seek critical health care.

### Promote Respect and Eliminate Stigma

The perception that people who use substances are less deserving of respect drives harmful policies, practices, language, and attitudes that create barriers to health for families with substance use. Health care workers can start by using language that builds and demonstrates respect for pregnant patients who use drugs. For example, “person with opioid use disorder” is respectful, while “addict” is stigmatizing. These, often unconscious, biases are disrupted when we follow the leadership of people with lived and living experience. We can begin with meaningful inclusion of people who use drugs in planning, implementation, and evaluation of individual care plans, healthcare practices, and health and social policy. One way to do this is by integrating peer support specialists and doulas with lived experience of substance use in pregnancy into perinatal care teams.^1^

### Support Patient Goals and Timelines

Many birthing people find it daunting to change their relationship with substances in the short timeframe of their pregnancy. Most of us benefit from setting small, achievable goals. For example, a parent might start by using human milk test strips for alcohol when they are drinking, rather than trying to become abstinent right away. Celebrating any positive change—no matter how small—can improve therapeutic relationships, build trust, and increase the self-efficacy and confidence that pregnant and early parenting people need to tackle subsequent goals. Finally, policymakers need to consider the nonlinear and individualized nature of recovery timelines, for example allowing parents more time to achieve stable recovery before terminating parental rights.

### Address the Social Determinants of Health and Root Causes of Perinatal Substance Use

Our relationship to substances is impacted by the environment in which we live, love, and work (Gage & Sumnall, 2019), as well as experiences of trauma (Marcellus, 2014). Cycles of trauma can be interrupted by access to the social determinants of health. Community programs that use a harm reduction framework provide not only prenatal care, but support for food, housing, employment, and other necessities (Streetworks, 2018). When we build communities with a focus on human connection, economic opportunity, recreational and cultural activities, we give people alternative methods to fill the needs that might otherwise be addressed by drugs. Social determinants of health could be improved for families with substance use by allowing parents with felony drug convictions to access the Supplemental Nutrition Assistance Program, the Temporary Assistance for Needy Families program, and extending Medicaid insurance coverage to at least one year postpartum to ensure continuous access to treatment medications beyond the immediate postpartum period.

## Advancing Harm Reduction Now and in the Future

To achieve equitable treatment for families with perinatal substance use, shared decision making and respect for autonomy and personhood is the starting point for health care workers. Every major medical, nursing, and public health group is a proponent of approaching substance use disorders in pregnancy as a public health concern and *not* a moral failing (Pregnancy Justice, 2021). Health care workers, especially nurses, are among the most trusted messengers in the United States. Our elected representatives, lawmakers, and especially our patients, are relying on us to inform law and policy built on evidence-based understanding of substance use and treatment. A critical component of a perinatal harm reduction framework is to create and implement supportive policies.

Systemic change happens when we identify intersections and build connections between harm reduction and other movements for social justice. When people who use drugs insist that their health conditions do not define their parenting ability, they draw on work from disability justice advocates. It is important to strive to correct the inequitable treatment of Black babies and their families affected by substance use by joining the larger movement for Black lives and Reproductive Justice. Reproductive freedom requires that we are never punished or controlled based on ostensible risks of what we put into our bodies. Perinatal harm reduction allows us to clear the barriers for people who use substances and turn healthy choices into easy choices.

## Footnotes

1 Project Nurture in Oregon is an example of a program that integrates peer doula support within existing systems of care.
